# Effect of excessive infant crying on resting BP, HRV and cardiac autonomic control in childhood

**DOI:** 10.1371/journal.pone.0197508

**Published:** 2018-05-31

**Authors:** Laetitia J. C. A. Smarius, Manon van Eijsden, Thea G. A. Strieder, Theo A. H. Doreleijers, Reinoud J. B. J. Gemke, Tanja G. M. Vrijkotte, Susanne R. de Rooij

**Affiliations:** 1 Department of Public Health, Amsterdam Public Health Research Institute, Academic Medical Center, University of Amsterdam, Amsterdam, The Netherlands; 2 Academic Center for Child and Adolescent Psychiatry de Bascule, Duivendrecht, The Netherlands; 3 Department of Child and Adolescent Psychiatry, VU University Medical Centre, Amsterdam, The Netherlands; 4 Department of Epidemiology, Health Promotion and Health Care Innovation, Public Health Service, Amsterdam, The Netherlands; 5 Arkin Institute for Mental Health, Amsterdam, The Netherlands; 6 Department of Pediatrics, VU University Medical Center, Amsterdam, The Netherlands; 7 Department of Clinical Epidemiology, Biostatistics and Bio-informatics, Academic Medical Center, University of Amsterdam, Amsterdam, The Netherlands; TNO, NETHERLANDS

## Abstract

**Objective:**

Early life stress has been shown to influence the developing autonomic nervous system. Stressors in infancy may program the autonomic nervous system resting state set point, affecting cardiovascular function in later life. Excessive crying may be an indicator of increased stress arousal in infancy. We hypothesized that excessive infant crying is related to altered cardiac autonomic nervous system activity and increased blood pressure at age 5–6 years.

**Methods:**

In the Amsterdam Born Children and their Development study, excessive crying, maternal burden of infant care and maternal aggressive behavior in the 13^th^ week after birth (range 11–16 weeks) were reported using questionnaires. Blood pressure, heart rate, heart rate variability and indicators of cardiac autonomic nervous system activity (sympathetic drive by pre-ejection period, parasympathetic drive by respiratory sinus arrhythmia) were measured at age 5–6 years during rest. Inclusion criteria were singleton birth, term-born, and no reported congenital or cardiovascular problems (N = 2153 included).

**Results:**

Excessive crying (2.8%) was not associated with resting heart rate, heart rate variability, pre-ejection period, respiratory sinus arrhythmia nor with blood pressure at age 5–6 years.

**Conclusions:**

Excessive infant crying as an indicator of increased stress arousal does not seem to be related to resting activity of the autonomic nervous system or blood pressure at age 5–6. Potential associations may become visible under stressed conditions.

## Introduction

During the first years of life the autonomic nervous system (ANS) is developing continuously: heart rate (HR) decreases, parasympathetic nervous system activation increases and sympathetic nervous system activation decreases, and frequency of classic reactivity profiles of reciprocal sympathetic activation and parasympathetic withdrawal increases [1]. Already in infancy, mental stress in children has been suggested to affect the resting state set point of the developing ANS. In 6-months old infants, the stress of high parental conflict was shown to be related to lower respiratory sinus arrhythmia (RSA) at baseline and during a stressful still face experiment [2]. Also, children enduring the stress of chronic pain had significantly lower resting heart rate variability (HRV) compared to healthy children [3]. On the long term, a harsh childhood family environment and negative emotionality have been shown to predict increased blood pressure (BP) after 10 years [4]. University students who lost a parent before the age of 16 or who reported poor quality of family relationships also showed higher BP [5].

Excessive infant crying (crying for three or more hours per 24 hours on average in the past week according to the mother at 3 months of age) can be considered an expression of increased stress arousal of the infant [6]. Whereas the usual reduction of crying after six weeks of age coincides with an increase in emotion control of the infant, for example the start of responsive or social smiling [7], excessive crying at age 3 months may indicate emotion regulatory difficulties. Excessive crying at 3 months was associated with a doubled risk of mood and behavioral problems at age 5–6 years [8]. As early life stress may shift the ANS balance of the child towards increased sympathetic activity and decreased parasympathetic activity and increase BP, excessive infant crying may be expected to be associated with altered ANS balance and elevated BP in later childhood. We would particularly expect that excessive infant crying is associated with decreased HRV as HRV is known to be associated with affect regulation and self-regulatory behavior [9,10].

Importantly, the infant-mother dyad can both contribute to decreasing stress and discomfort in the infant, as well as contribute to increasing stress, possibly affecting ANS balance and blood pressure in the child. Postnatal maternal psychiatric symptoms have been shown to be associated with higher mean HR and lower vagal modulation in children at 14 months of age [11]. On the other hand, high maternal sensitivity has been shown to be associated with higher levels of infant HRV [12]. Additionally, infants of mothers with previous prenatal stress cried less when their mother had a high level of self-efficacy compared to infants whose mother had low self-efficacy [13]. Maternal stress might influence the developing autonomic nervous system in the child, thus can be a considered a confounder. The aim of this study is to investigate prospectively the association between excessive infant crying in infancy and the child’s cardiac ANS and BP at rest in young childhood, hereby adjusting for important mother-infant dyad aspects.

## Methods

The present study is part of a large prospective, observational, population-based multiethnic birth cohort, the Amsterdam Born Children and their Development (ABCD) study. Extensive information about the cohort and procedures regarding data collection is provided elsewhere [14]. Approval of the study was obtained from the Central Committee on Research involving Human Subjects in The Netherlands, the Academic Medical Center Medical Ethical Committee, the VU University Medical Center Medical Ethical Committee and from the Registration Committee of the Municipality of Amsterdam. All participating mothers gave written informed consent for themselves and their children. Data are from the ABCD cohort study. These data are not available for a public repository for ethical reasons. These data restrictions were specifically imposed by the medical ethics committee of the Academic Medical Centre in Amsterdam. However, the ABCD study can be contacted for requests to access the data (abcd@amc.uva.nl).

**Fig 1:**
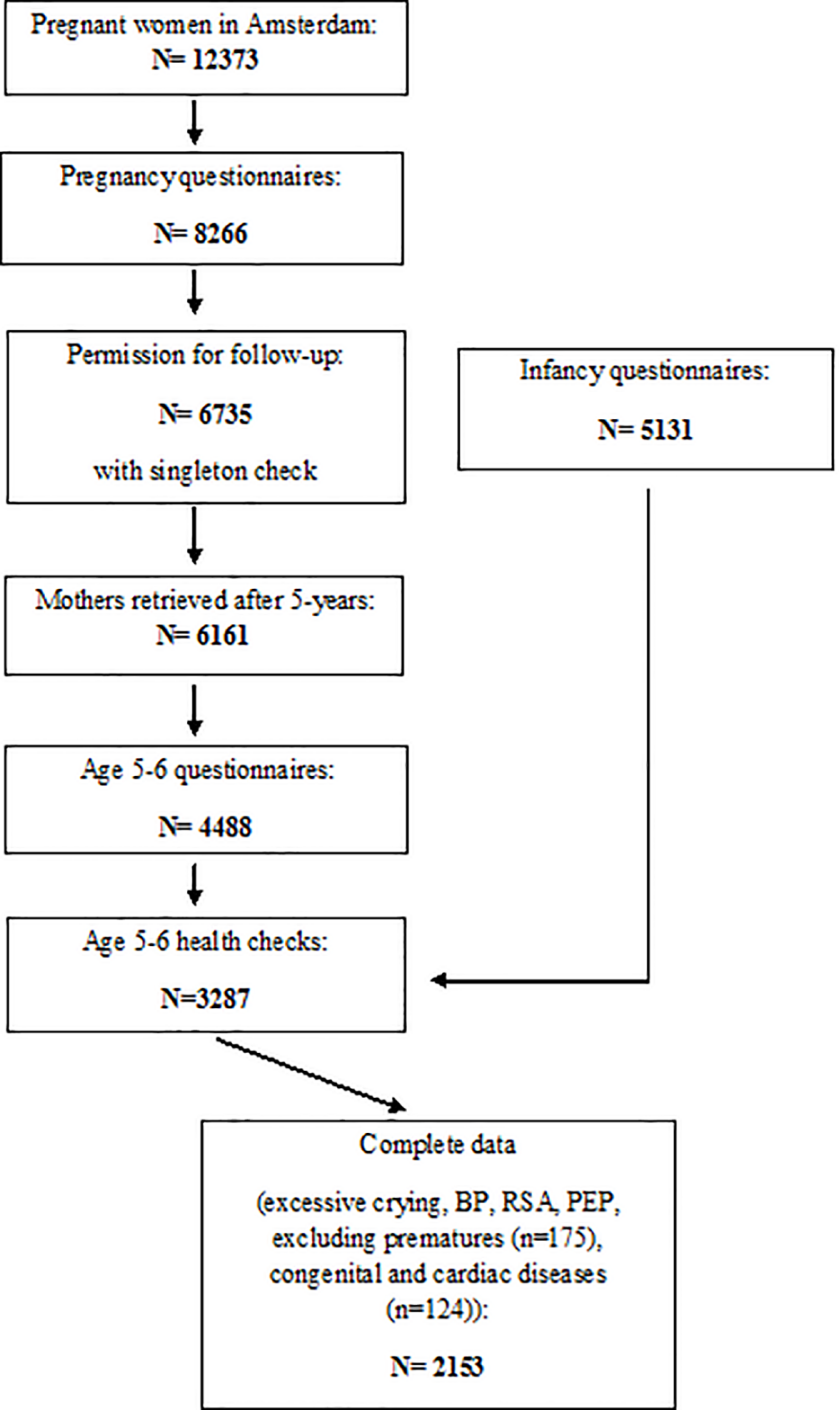
Flow chart of participants included for analysis.

In 2003 and 2004, 12,373 pregnant women who first attended antenatal care in Amsterdam were approached to participate in the ABCD study. Of these women, 8,266 (67%) returned the pregnancy questionnaire (phase 1). Anonymized record linkage of cohort study data with national registry data indicated that selective non-response was present in the ABCD-study, but selection bias was acceptably low. The ABCD non-respondents were significantly younger, more often non-western, and more often multiparae [15]. Three months after birth, the infancy questionnaire was sent out to the women who gave permission for follow-up (n = 6,735) (phase 2). For the questionnaire at the child’s age of 5–6 years, 6,161 mothers were retrieved from the Youth Health Care Register (phase 3). The mothers received a questionnaire, including an informed consent form for the age five health check, which was returned by 4,488 (73%) of the participants. Attrition in this follow-up number was due to withdrawal, unknown address, infant or maternal death. The health check itself consisted of various health measurements in 3,287 children aged 5–7 years (2008–2010; mean age 5.7 years, SD 0.5). Multiple births, preterm births and congenital disorders were excluded from the cohort. Mother-child dyads of which the questionnaire was filled out after the infant’s age of 16 weeks were excluded as well. The selection of the population included in the current study’s analyses (N = 2,153) is visualized in Fig 1.

### Excessive crying in infancy

Information on infant excessive crying was obtained from items in the infancy questionnaire that was completed in the 13^th^ week after birth (range 11–16 weeks). Mothers were asked to estimate the number of hours their baby cried on average, over each 24 hour period in the past week. Mothers reported the amount of hours their baby was crying on a 9-point scale. They could choose from less than one hour (1) to 8 hours or longer (9). Excessive crying was defined as crying for three or more hours per 24 hours on average in the past week according to the mother (best approximation of the Wessel’s criteria [16]). As such, this definition was previously used by the ABCD research group and shown to be related to antenatal stress levels and mood and behavioral problems at age 5–6 [8,17].

### Resting cardiac autonomic nervous system activity and blood pressure of the child

**Fig 2:**
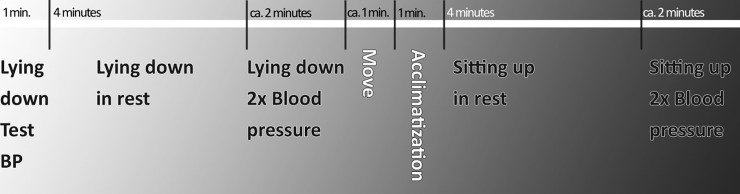
Recording schedule of resting cardiac autonomic nervous system activity and blood pressure.

Cardiac ANS activity was assessed at the age five health check, using an ambulatory device, the VU University Ambulatory Monitoring System (VU-AMS, version 5fs, TD-FPP, Amsterdam, the Netherlands) [18]. Reliability and validity aspects and recording methodology of the VU-AMS have been described previously [19,20]. The system records three lead electro cardiograms (ECG) and four lead impedance cardiograms (ICG) (Ultratrace Diagnostic ECG with wet gel; ConMed Corporation, Utica, New York, United States of America). The procedure of this measurement has been extensively described previously [21]. In short, ECG and respiratory activity were recorded during the day between 8:30 am and 16:30 pm in supine and sitting positions. This was done in order to reduce the effects of the circadian rhythm. During the recording periods the children were accompanied by the researcher only and no conversation took place. The children were listening to an audiobook during the whole 14 minutes recording period. The recording schedule is shown in Fig 2. RR-interval time series were analyzed during 4 minutes. Mean HR was 85.5 bpm (60 sec/85.5 = 0.7 sec for RR-interval). All R-peaks in the ECG were checked and R-peak markers were moved, inserted or deleted. Premature ventricular complexes, that occurred infrequently, and movement artefacts were deleted. No interpolation was performed. The VU-DAMS software [22] marked inspirations and expirations in the respiratory signals, which were manually checked, and no edits were necessary [23]. Inspiration-Expiration difference was averaged over all cycles of respiration in the 4 minutes of registration. The classical parameters of HRV, the frequency parameters of the Low Frequency (LF) and High Frequency (HF), and their normalized values, were obtained as well.

RSA, a time domain index of HRV in the respiratory frequency range, has been automatically obtained as derivate of parasympathetic nervous system activity. RSA was taken as the peak valley estimation (pvRSA) which had been automatically obtained by subtracting the shortest inter beat interval during HR acceleration in the inspirational phase from the longest inter beat interval during deceleration in the expirational phase [18] Decreased parasympathetic activity is indicated by a shorter RSA. Pre-ejection period (PEP) is a measure of sympathetic activity. PEP can reliably be measured in children [24]. PEP is the time interval between the onset of the ventricular depolarization (Q wave onset in the ECG) and the time of opening of the aortic valves (B point in the ICG) and was scored manually in large-scale ensemble averages of the impedance cardiograms. Decreased sympathetic activity is indicated by a longer PEP [25]. To obtain the mean value of the HR, the mean HR across all the experimental conditions was used, excluding the period during which BP was measured.

BP was measured with the automatic oscillometric method, using the Omron 705 IT (Omron Healthcare Inc., Bannockburn, IL, USA) with a small cuff (arm circumference 17–22 cm) on the non-dominant arm. After resting in the supine position for four minutes, systolic (SBP) and diastolic blood pressure (DBP) were measured twice. After resting in the sitting position for four minutes, systolic (SBP) and diastolic blood pressure (DBP) were measured twice again. When either the SBP or DBP differed more than 10 mmHg between the two measurements, a third measurement was taken (n = 502: 17%). The average of the two measurements closest together was used. The recording schedule of resting cardiac ANS activity and BP is visualized in Fig 2.

### Covariates

The following maternal characteristics were included: age, parity (0 or > = 1), country of birth (industrialized country or non-industrialized country), cohabitation status (single or living together with partner), level of education (years after primary school) as a measure of socioeconomic status, maternal pre-pregnancy BMI (kg/m2), and pre-pregnancy hypertension (yes or no). Pregnancy-induced hypertension (yes or no) was available by combining data from the questionnaire and Dutch Perinatal Registration (PRN, www.perinatreg.nl) and classified in accordance with the guidelines of the International Society for the Study of Hypertension in Pregnancy (www.isshp.com). Paternal hypertension and family hypertension were derived from a questionnaire previously described elsewhere [26]. Other maternal characteristics obtained from the infancy questionnaire were: maternal smoking at home (yes or no), and maternal depressive symptoms. Maternal depressive symptoms during infancy were measured using the Center for Epidemiologic Studies Depression Scale (CES-D) [27,28], the summed scores were dichotomized (<16 or > = 16), using the cut-off for possible cases. Maternal burden of infant care was measured using five questions on a four point scale (very true, true, not true, not true at all): ‘all things considered, taking care of my baby is not so hard; taking care of my baby is quite a burden to me; the care of my baby takes up so much of my energy that other family members are neglected; my baby is not easy to take care of; taking care of my baby is too demanding for me’. The summed scores were measured continuously. Maternal aggressive behavior (coercive interaction) during infancy was assessed by the following four questions: ‘did you speak angry to stop the baby’s crying?; did you use your hand or a cloth to stop baby’s crying?; did you slap the baby to stop the crying?; did you shake the baby to stop crying?’ and consisted of four behaviors which were dichotomized [angry speaking (frequency < = 1 or > = 2), cloth on mouth (frequency 0 or > = 1), slapping (frequency 0 or > = 1), shaking baby (frequency 0 or > = 1)]. These last three behaviors were scored as physical aggression if one out of three was present or not (yes or no). Angry speaking (yes or no) and physical aggression (yes or no) were subsequently combined into one variable: maternal aggressive behavior (yes or no). Maternal aggressive behavior was considered present if either angry speaking or physical aggression was present [8]. Both correlations between burden of infant care and maternal aggressive behavior, as well as between burden of infant care and maternal depressive symptoms were low, respectively: r = 0.20 (p<0.001) and r = 0.29 (p<0.001), which supports their differences in construct. The following child characteristics were included: sex, birth weight, gestational age, and exact age at the health check at 5–6 years. The height and weight of the child were measured at the age 5 health check from which BMI was calculated.

### Data analyses

Analyses were conducted using SPSS 19.0 (SPSS inc, Chicago, IL, USA). All variables were inspected on outliers and checked on normality. Descriptive statistics were used to describe maternal and child characteristics and excessive crying; statistical differences were tested using analysis of variance for continuous variables and Chi square tests for categorical variables. As the distribution of the HF and LF was highly skewed, these outcome data were log transformed. The associations between excessive crying and HR, resting cardiac ANS activity, HF, LF, and resting BP were analyzed by means of multiple linear regression analysis. All potential confounders were selected a priori and included in the regression model by using a forced- entry method. We selected confounders which were significantly associated with excessive infant crying. In the first model, associations were adjusted for sex, age and height of the child. Subsequently, the potential confounders birth weight, maternal age, maternal education, ethnicity, maternal pre-pregnancy BMI, maternal burden of infant care, maternal aggressive behavior (verbal or physical), maternal depressive symptoms in infancy, and family hypertension were added to a second model. The significance level we used in the study was 5%.

## Results

### Subjects characteristics

The characteristics of both the mothers and the children are presented in Table 1. Of the 2153 included children, 61 (2.8%) had a history of excessive crying at the age of three months. Compared to non-excessive crying infants, excessive crying infants more often had a slightly lower birth weight, a lower educated mother, a mother who was slightly younger, was born in a non-industrialised country, had a higher pre-pregnancy BMI, reported higher levels of depressive symptoms during infancy, experienced higher burden of infant care, and more often self-reported use of aggressive behaviour. Their families suffered more often from hypertension.

**Table 1:** Demographic characteristics of 2153 women and their children according to excessive crying status (N = 61).

	N	%or mean (SD)	Excessive cryingN = 61 (2.8%) % or mean (SD)	Non excessive crying N = 2092 (97.2%) % or mean (SD)	*p*
**Child characteristics at birth**	2153				
Gender: female	1080	50.2%	55.7%	50.0%	0.38
Birth weight, grams, mean (SD)	2147	3543 (490)	3386 (541)	3547 (488)	0.01
Gestational age, wk, mean (SD)	2153	39.7 (1.2)	39.4 (1.2)	39.7 (1.2)	0.05
**Maternal characteristics**					
Pre-pregnancy BMI, mean (SD)	2153	22.9 (3.6)	23.9 (3.8)	22.8 (3.6)	0.02
Pre-pregnancy hypertension	53	2.5%	3.3%	2.4%	0.68
Pregnancy related hypertension	206	9.6%	8.2%	9.7%	0.70
Primipara	1209	56.2%	54.1%	56.2%	0.74
Maternal age, years, mean (SD)	2153	32.1 (4.5)	29.6 (4.9)	32.2 (4.4)	<0.001
Cohabitancy: living with partner	1953	90.8%	85.2%	91.0%	0.13
Education, years after primary school, mean (SD)	2143	10.0 (3.5)	7.8 (4.0)	10.1 (3.4)	<0.001
Ethnic background:					0.002
Industrialized	1818	84.4%	70.5%	84.8%	
Non-industrialized	335	15.6%	29.5%	15.2%	
**Maternal factors in infancy**					
Maternal burden of infant care	2153	8.2 (2.3)	10.1 (2.5)	8.2 (2.3)	<0.001
Maternal depression (CES-D)	283	13.2%	34.4%	12.5%	<0.001
Maternal aggressive behavior	244	11.3%	19.4%	11.1%	0.04
Maternal smoking at home	77	3.6%	6.6%	3.5%	0.20
**Child’s preschooler period**					
Age of the child, mean (SD)	2146	5.2 (0.3)	5.3 (0.4)	5.2 (0.3)	0.001
Child height at age 5, mean (SD)	2153	116.6 (5.8)	116.9 (6.4)	116.6 (5.7)	0.66
Child BMI at age 5, mean (SD)	2153	15.5 (1.4)	15.7 (1.5)	15.5 (1.4)	0.28
Respiration rate	2153	19.1 (2.2)	19.2 (2.6)	19.1 (2.2)	0.87
**Family hypertension**					
Paternal hypertension	100	4.7%	0.0%	4.8%	0.09
Family (M or P family) hypertension	129	6.3%	14.8%	6.1%	0.010

### Association between excessive crying in infancy and children’s ANS and blood pressure at age 5–6

Excessive infant crying was not associated, univariate nor multivariate, with an altered resting state ANS activity or HRV nor with an increased HR or SBP or DBP at the age of 5–6 (Table 2). Subsequent analysis based on continuous data investigating crying, in hours per day, in relation to cardiovascular outcomes at age 5–6 also showed no significant associations (S1 Table).

**Table 2:** Differences in BP, HR, HRV and resting ANS activity at age 5–6 years according to excessive crying in early infancy compared to non-excessive infant crying.

	Excessive cryingNo N	Mean(SD)	Excessive cryingYes N	Mean(SD)	Difference B (95%CI)	Model 1 Adjusted difference B (95%CI)	Model 2 Adjusted difference B (95%CI)
SBP (mmHg)							
supine	2092	99.3 ± 7.2	61	100.3 ± 6.4	1.0 (-0.9,2.8)	1.1 (-0.8,3.0)	0.1 (-1.8,2.0)
sitting	2092	97.7 ± 8.6	61	97.9 ± 6.6	0.1 (-2.0,2.3)	0.0 (-2.2,2.3)	-1.2 (-3.4,1.1)
DBP (mmHg)							
supine	2092	57.1 ± 5.7	61	58.2 ± 5.2	1.1 (-0.3,2.6)	1.2 (-0.3,2.7)	0.3 (-1.2,1.8)
sitting	2092	58.0 ± 7.9	61	59.6 ± 5.1	1.6 (-0.4,3.5)	1.8 (-0.3,3.8)	0.9 (-1.2,3.0)
HR (bpm)							
supine	2092	85.4 ± 9.8	61	85.2 ± 9.4	-0.2 (-2.7,2.3)	0.4 (-2.2,2.9)	-0.3 (-2.9,2.3)
sitting	1964	91.0 ± 10.1	58	90.2 ± 9.5	-0.8 (-3.4,1.8)	0.0 (-2.7,2.7)	-0.5 (-3.3,2.3)
PEP (msec)							
supine	2092	71.2 ± 9.0	61	71.6 ± 8.5	0.4 (-1.9,2.7)	0.1 (-2.3,2.5)	0.0 (-2.4,2.5)
sitting	2074	72.9 ± 11.7	60	74.6 ± 13.1	1.7 (-1.3,4.6)	1.1 (-2.1,4.3)	1.1 (-2.1,4.3)
RSA (msec)							
supine	2092	124.1 ± 58.4	61	125.5 ± 66.2	1.4 (-13.5,16.3)	-1.3 (-17.2,14.6)	0.1 (-16.0,16.2)
sitting	2050	111.2 ± 52.8	59	111.8 ± 49.6	0.6 (-13.0,14.3)	-2.1 (-16.7,12.5)	-2.1 (-17.0,12.8)
ln HF							
supine	2081	7.3 ± 1.2	61	7.2 ± 1.4	-0.1 (-0.4,0.2)	-0.1 (-0.5,0.2)	-0.1 (-0.4,0.2)
sitting	2020	7.1 ± 1.1	60	7.0 ± 1.3	-0.1 (-0.4,0.2)	-0.1 (-0.4,0.2)	-0.0 (-0.3,0.3)
ln LF							
supine	2080	6.7 ± 0.8	61	6.6 ± 0.9	-0.1 (-0.3,0.1)	-0.1 (-0.3,0.1)	-0.1 (-0.3,0.1)
sitting	2019	6.7 ± 0.8	60	6.7 ± 0.9	-0.1 (-0.3,0.1)	-0.0 (-0.2,0.2)	-0.0 (-0.2,0.2)

Model 1: Multiple linear regression analyses, adjusted for sex, height and age of the child; Model 2: Model 1, additionally adjusted for birth weight, maternal age, maternal education, ethnicity, maternal pre-pregnancy BMI, maternal burden of infant care, maternal aggressive behavior (verbal or physical), maternal depressive symptoms in infancy, and family hypertension.

B = Unstandardized regression coefficient. 95%CI = 95% Confidence Interval for B.

## Discussion

We did not find evidence for our hypothesis that excessive crying babies develop alterations in resting ANS activity and an increased SBP and DBP in later childhood. To the best of our knowledge, we are the first to examine the association between excessive crying in infancy, as an early stress arousal indicator, and resting state ANS activity or HRV and BP at age 5–6. Our findings regarding ANS activity are in line with earlier results from a previous study of the ABCD cohort in which the effect of maternal prenatal psychosocial stress on the child’s ANS were investigated [29]. Neither maternal prenatal psychosocial stress, nor excessive crying in infancy as an indicator of increased stress arousal seems to deregulate cardiac ANS balance in rest when the child reaches the age of 5–6 years.

A number of strengths and limitations of our study have to be considered. Strong points include that we were able to control for a large number of important potential confounding factors, for example ethnic background and maternal depressive symptoms. We specifically excluded participants with previously known physiological variables of influence on the ANS and BP such as preterm birth. Preterm birth and fetal growth restriction have been shown to be associated with increased sympathico-adrenal activity in childhood, as indicated by stress-induced increases in HR and urinary catecholamines [30]. Also, we used a highly standardized protocol for measuring ANS activity and BP and a previous study in this cohort showed significant associations between our ANS variables and metabolic outcomes at age 5–6 years [31].

There were also limitations. We did not use the generally accepted Wessel’s definition of excessive infant crying (crying >3 hours a day for > 3 days in 3 consecutive weeks) [16], but its best approximation based on the available data. However, excessive infant crying according to Wessel’s definition was shown to occur in 2–2.5% of the Dutch infant population [32–34], which is very similar to the prevalence in our study of 2.8% suggesting comparable constructs. Importantly, most babies are known to show a reduction of crying from 6 weeks onwards [35]. Although we relied solely on maternal report of excessive crying, as paternal report on excessive crying was not collected, we do not expect this limitation to lessen the validity of our findings, because ratings of excessive crying were shown not to differ between fathers and mothers in another cohort study, suggesting that parents have similar judgments or perceptions of the hours of crying of their infant [36].

The current study was conducted in a large, prospective, population-based, multi-ethnic birth cohort. As in most cohort studies, selective loss to follow-up was present. The present subgroup is a slightly healthier reflection of the total ABCD population (i.e. higher educational level, lower child BMI). Additionally, the low prevalence of excessive crying, although in line with the prevalence in the general population, did limit our power to detect differences between groups. Nevertheless, a subsequent analysis based on continuous data investigating crying (hours/day) in relation to cardiovascular outcomes at age 5–6 also showed no significant associations.

We did not have data on child attachment and child adversities, including family environment or parental conflict, in the period between infancy and the preschool age. These factors have been shown to be associated with ANS and BP [37,38] and could differ between excessive crying infants and non-excessive crying infants. For example, infants’ avoidant attachment has been associated with lower HR and higher RSA at the age of 4 years [36]. Higher levels of maternal postnatal depressive symptoms have been shown to be associated with lower resting RSA in disorganized attached infants, but not in non-disorganized attached infants after a slightly shortened Strange Situation paradigm at 14 months [36]. Adjustment for these attachment styles might have influenced our results. However, as in the general population most infants (66%) would develop an organized attachment style [38] we do not expect this to have altered our findings. Moreover, excessive infant crying occurs before the child’s preferred attachment style has evolved and excessive crying disappears most often before the age of 4 months [39,40].

Our data suggest that excessive crying at age 3 months in itself is not a risk factor for the development of altered resting ANS activity, HRV or increased BP. However, this leaves open the possibility that the, in her first year, insecure attaching young child, with a more generalized ‘persistent mother-infants distress syndrome’ after the first 4 months [39,41] could be at higher risk for an altered resting ANS, HRV or altered BP. The distinction between temporary and persistent distress in the child could not be made based on our data, but may explain our null-finding.

Another explanation for our null-finding could be that we measured the children at rest. It is possible that excessive crying as an indicator of stress arousal is related to the set points for stress reactivity. Generally, differences in cardiovascular function are more likely to become apparent when the system is stressed. For example, in stress reactivity research in infants, infants exposed to anger showed greater RSA withdrawal to mothers' still-face than infants exposed to other emotions. ‘Exposure to anger may sensitize infants to stress and lead to increased need for physiological regulation’[42]. Stress reactivity was beyond the scope of our study. Future studies on excessive infant crying should consider stress reactivity during childhood.

An association between excessive infant crying and cardiovascular outcomes at age 5–6 might exist in subgroups, for example in a group of excessive crying infants with mothers who show aggressive behavior to their infants. Due to the small number of cases (both excessive infant crying and maternal aggressive behavior: n = 12) we were not able to study possible moderation by maternal aggressive behavior. We would recommend future studies to take into account several stress-related factors in childhood (child attachment, child or family adversity, family psychosocial stress, parental conflict) while investigating the association between excessive infant crying and ANS activity, HRV, HR and BP in childhood.

Finally, although we previously showed associations between excessive infant crying and mood and behaviorial problems at age 5–6 [8] and hypothesized that it may also affect biobehavioral regulation, our hypothesis may very well be false and associations between excessive infant crying and cardiovascular outcomes may not exist. As this was the first study on a potential association, future studies should confirm whether this is indeed the case.

Concluding, excessive infant crying in term born babies, at the age of 3 months, is neither associated with altered resting state ANS activity nor with an increased HR or BP at the age of 5–6. Our findings suggest no increased cardiovascular risk after excessive infant crying, an indicator of increased stress arousal, at young age.

## Supporting information

S1 Table Differences in BP, HR, HRV and resting ANS activity at age 5–6 years according to crying (hours/day) in early infancy. (DOCX)

## Abbreviations

BP: blood pressure
DBP: diastolic blood pressure
SBP: systolic blood pressure
HR: heart rate
BMI: body mass index
HRV: heart rate variability
LF: Low Frequency
HF: High Frequency
ANS: autonomic nervous system
PEP: pre-ejection period
RSA: respiratory sinus arrhythmia
pv RSA: peak valley estimation
ABCD: Amsterdam Born Children and their Development
VU-AMS: VU University Ambulatory Monitoring System
ECG: electro cardiograms
ICG: impedance cardiograms
PRN: Dutch perinatal registration
CES-D: Centre for Epidemiologic Studies Depression Scale
