# Supplementary material for: Effect of excessive infant crying on resting BP, HRV and cardiac autonomic control in childhood
Source: PLoS One. 2018 May 31;13(5):e0197508. doi: 10.1371/journal.pone.0197508 (PMC5979019; doi:10.1371/journal.pone.0197508)
Supplement: S1 Table — (DOCX) [file pone.0197508.s001.docx]

**S 1 Table**

*Differences in BP, HR, HRV, and resting ANS activity at age 5-6 years according to*

*crying (hours/day) in early infancy*

|  | Crude Model Difference B (95%CI) | Model 1 Adjusted difference B (95%CI) | Model 2 Adjusted difference B (95%CI) |
| --- | --- | --- | --- |
| SBP (mmHg)  supine  sitting | 0.1 (-0.3,0.4)  0.1 (-0.3,0.5) | 0.2 (-0.2,0.6)  0.2 (-0.2,0.6) | -0.1 (-0.5,0.3)  -0.1 (-0.6,0.3) |
| DBP (mmHg)  supine  sitting | 0.1 (-0.3,0.4)  0.0 (-0.3,0.4) | 0.2 (-0.1,0.5)  0.1 (-0.3,0.5) | -0.1 (-0.4,0.2)  -0.1 (-0.5,0.3) |
| HR (bpm)  supine  sitting | -0.1 (-0.5,0.4)  -0.2 (-0.7,0.3) | -0.0 (-0.4,0.5)  -0.1 (-0.6,0.5) | -0.1 (-0.6,0.4)  -0.2 (-0.8,0.3) |
| PEP (msec)  supine  sitting  RSA (msec)  supine  sitting  Ln HF  supine  sitting  Ln LF  supine  sitting | 0.0 (-0.4,0.5)  0.1 (-0.5,0.6)  -1.6 (-4.5,1.2)  -0.4 (-3.0,2,2)  -0.0 (-0.1,0.0)  -0.0 (-0.1,0.0)  -0.0 (-0.1,0.0)  -0.0 (-0.1,0.0) | 0.1 (-0.4,0.5)  0.1 (-0.5,0.7)  -2.1 (-5.2,0.9)  -0.8 (-3.6,1.9)  -0.0 (-0.1,0.0)  -0.0 (-0.1,0.0)  -0.0 (-0.1,0.0)  -0.0 (-0.1,0.0) | 0.0 (-0.5,0.5)  0.0 (-0.6,0.6)  -1.9 (-5.1,1.3)  -1.0 (-4.0,1.9)  -0.0 (-0.1,0.0)  -0.0 (-0.1,0.0)  -0.0 (-0.1,0.0)  -0.0 (-0.1,0.0) |

Model 1: Multiple linear regression analyses, adjusted for sex, height and age of the child;

Model 2: Model 1, additionally adjusted for birth weight, maternal age, maternal education,

ethnicity, maternal pre-pregnancy BMI, maternal burden of infant care, maternal aggressive

behavior (verbally or physically), maternal depressive symptoms in infancy, and family hypertension.

B= Unstandardized regression coefficient. 95%CI= 95% Confidence Interval for B.
